# Supplementary material for: Impact of Cesarean Delivery and Breastfeeding on Secretory Immunoglobulin A in the Infant Gut Is Mediated by Gut Microbiota and Metabolites
Source: Metabolites. 2023 Jan 18;13(2):148. doi: 10.3390/metabo13020148 (PMC9959734; doi:10.3390/metabo13020148)
Supplement: Supplementary file 1 [file metabolites-13-00148-s001.zip › Supplementary figures.pptx]

## Slide 1
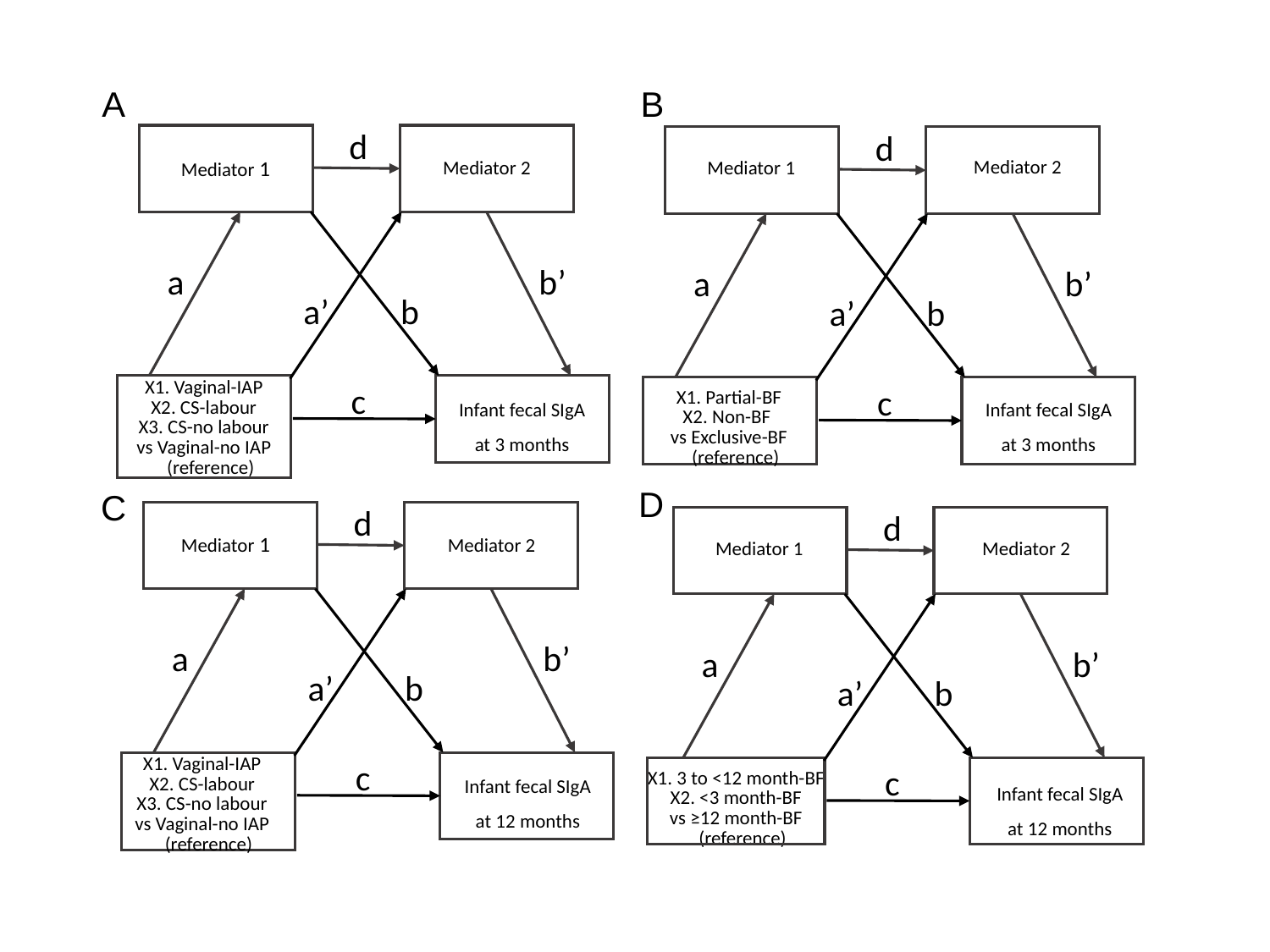

A
B
d
a
b’
a’
b
c
Infant fecal SIgA
at 3 months
Mediator 1
X1. Vaginal-IAP
X2. CS-labour
X3. CS-no labour
vs Vaginal-no IAP
 (reference)
d
a
b’
a’
b
c
Mediator 2
Mediator 1
Mediator 2
Infant fecal SIgA
at 3 months
X1. Partial-BF
X2. Non-BF
vs Exclusive-BF
 (reference)
D
C
d
a
b’
a’
b
c
Infant fecal SIgA
at 12 months
X1. Vaginal-IAP
X2. CS-labour
X3. CS-no labour
vs Vaginal-no IAP
 (reference)
d
a
b’
a’
b
c
Infant fecal SIgA
at 12 months
X1. 3 to <12 month-BF
X2. <3 month-BF
vs ≥12 month-BF
 (reference)
Mediator 1
Mediator 2
Mediator 1
Mediator 2

## Slide 2
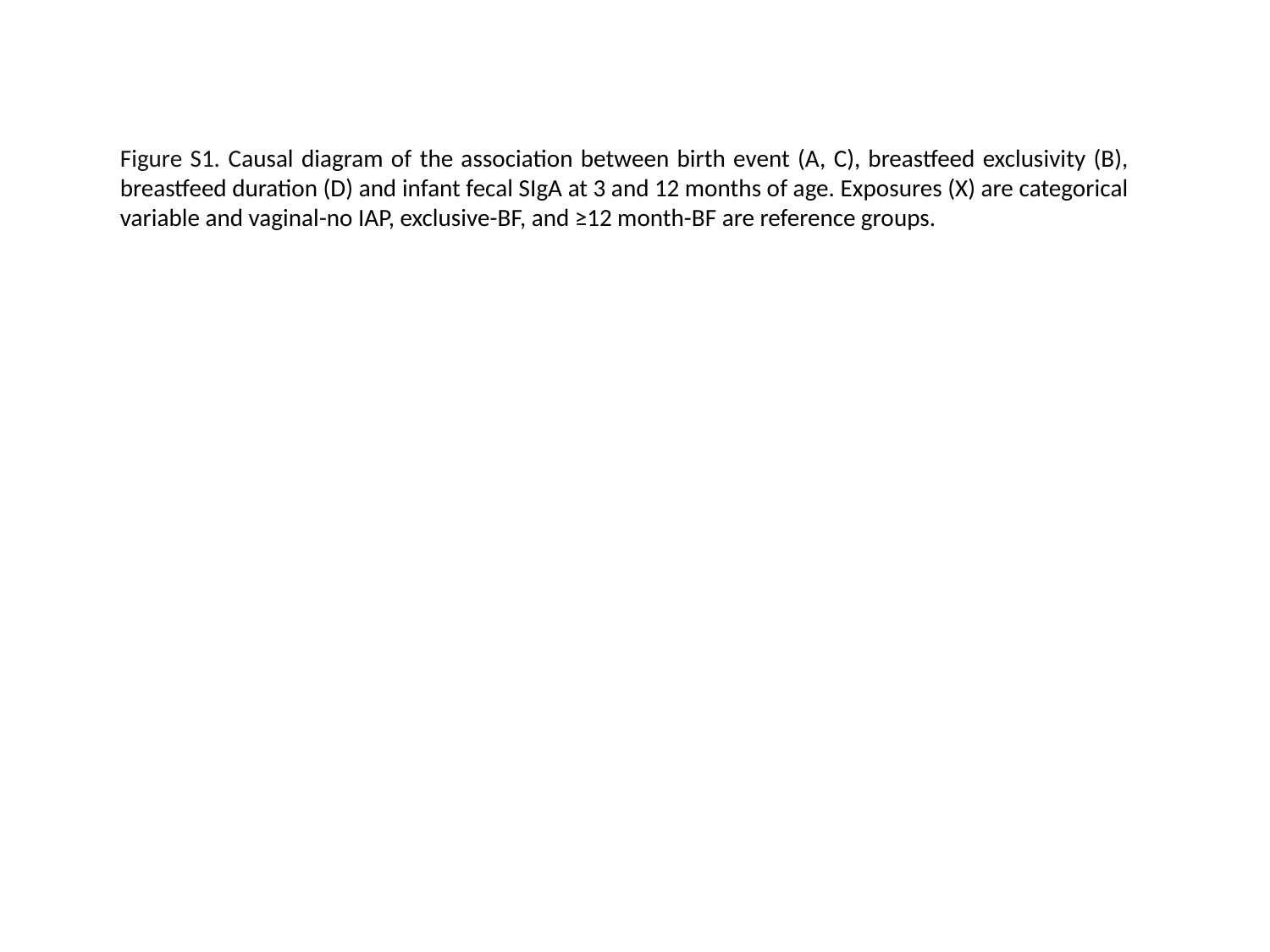

Figure S1. Causal diagram of the association between birth event (A, C), breastfeed exclusivity (B), breastfeed duration (D) and infant fecal SIgA at 3 and 12 months of age. Exposures (X) are categorical variable and vaginal-no IAP, exclusive-BF, and ≥12 month-BF are reference groups.

## Slide 3
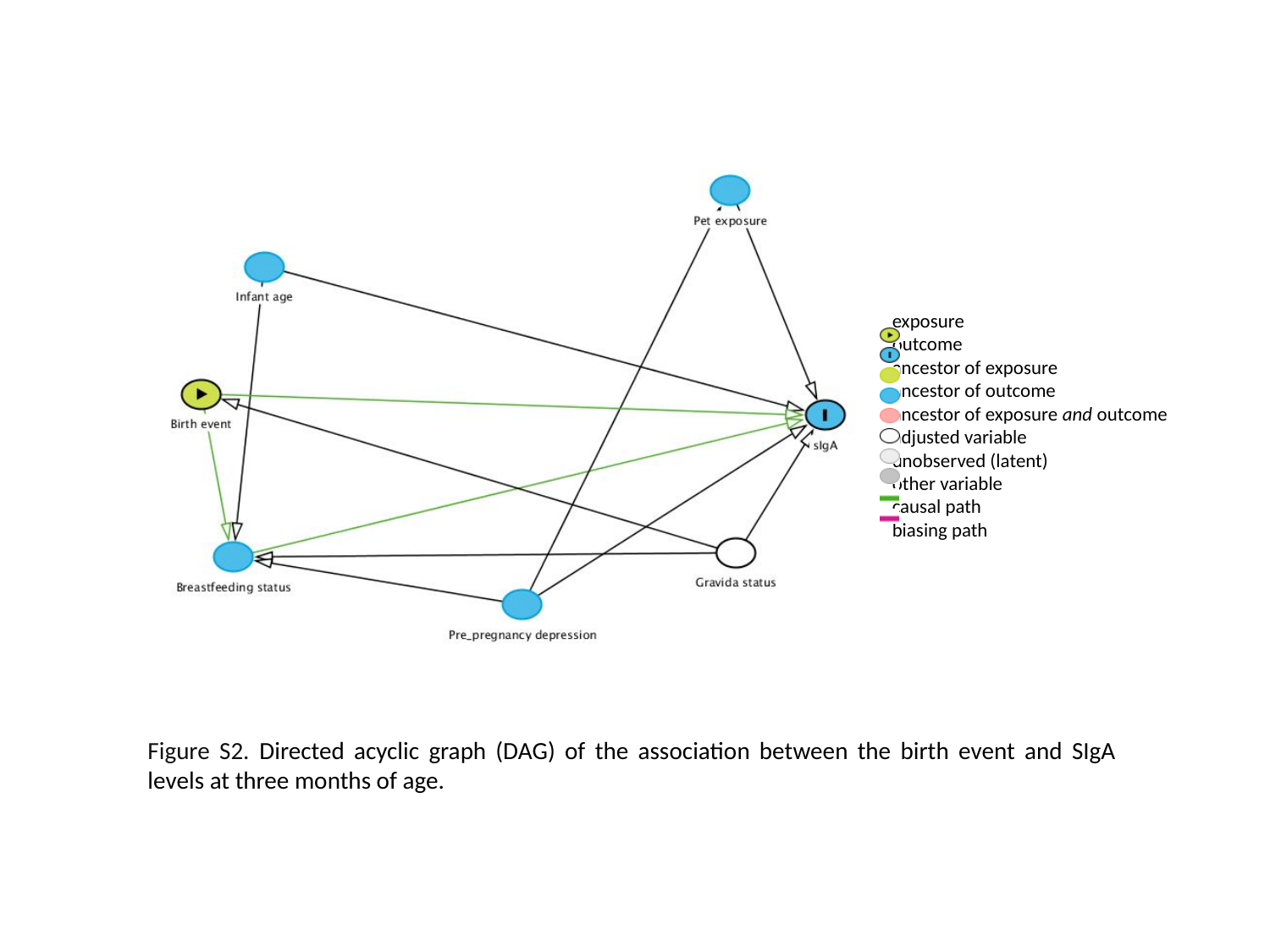

exposure
  outcome
  ancestor of exposure
  ancestor of outcome
  ancestor of exposure and outcome
  adjusted variable
  unobserved (latent)
  other variable
  causal path
  biasing path
Figure S2. Directed acyclic graph (DAG) of the association between the birth event and SIgA levels at three months of age.

## Slide 4
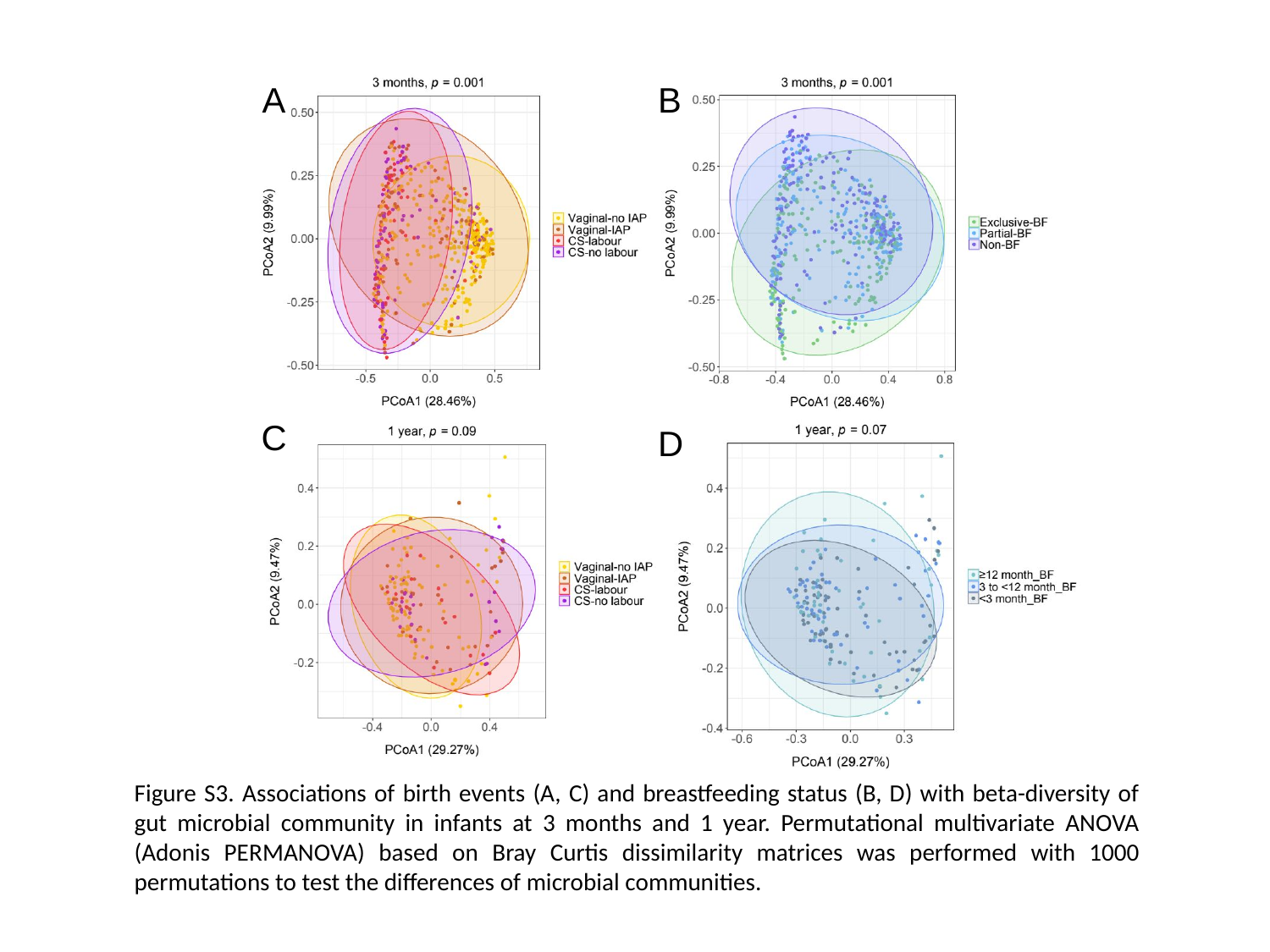

A
B
C
D
Figure S3. Associations of birth events (A, C) and breastfeeding status (B, D) with beta-diversity of gut microbial community in infants at 3 months and 1 year. Permutational multivariate ANOVA (Adonis PERMANOVA) based on Bray Curtis dissimilarity matrices was performed with 1000 permutations to test the differences of microbial communities.

## Slide 5
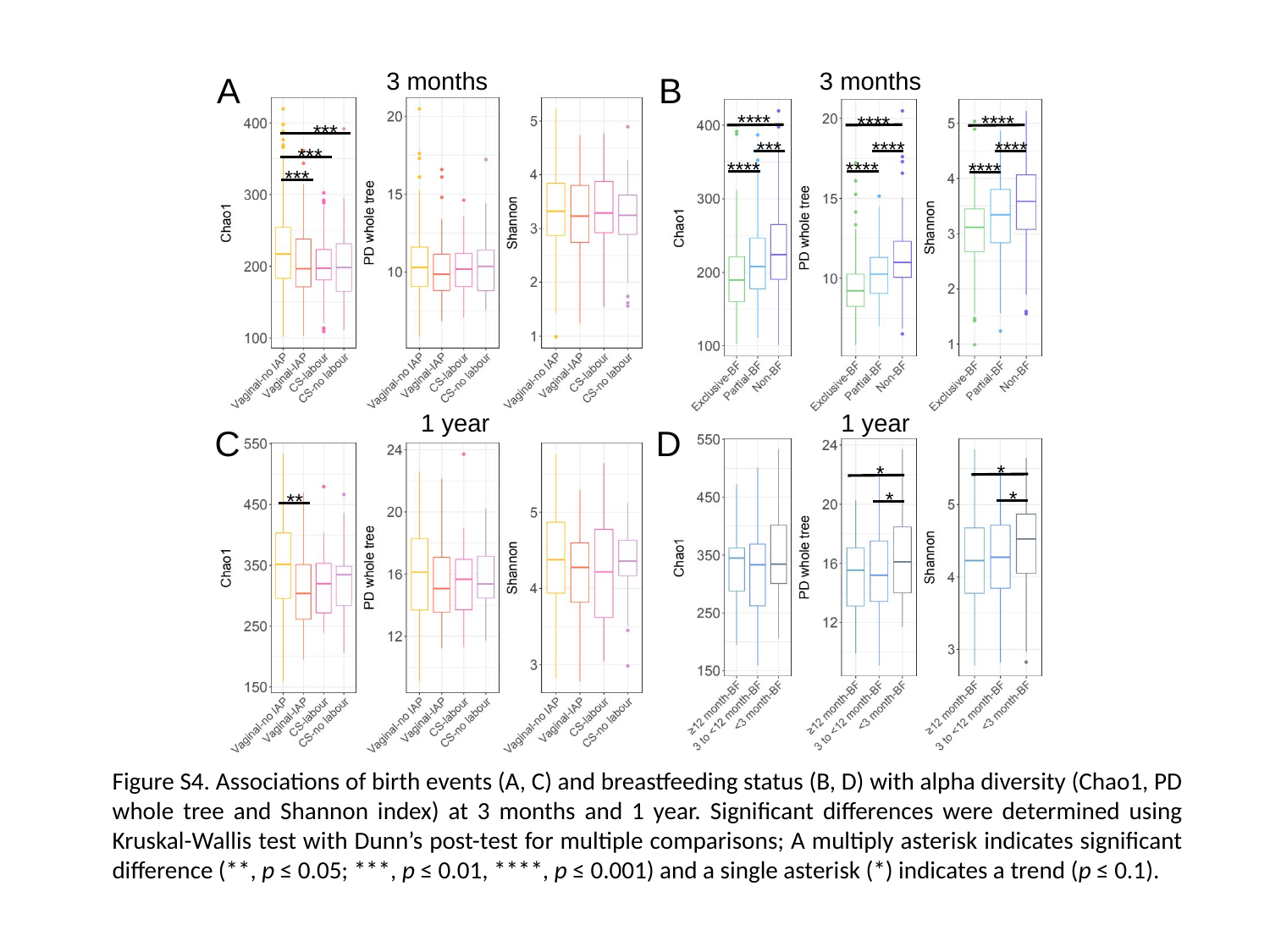

3 months
3 months
A
B
****
****
****
***
***
****
****
***
****
****
****
***
1 year
1 year
C
D
*
*
*
*
**
Figure S4. Associations of birth events (A, C) and breastfeeding status (B, D) with alpha diversity (Chao1, PD whole tree and Shannon index) at 3 months and 1 year. Significant differences were determined using Kruskal-Wallis test with Dunn’s post-test for multiple comparisons; A multiply asterisk indicates significant difference (**, p ≤ 0.05; ***, p ≤ 0.01, ****, p ≤ 0.001) and a single asterisk (*) indicates a trend (p ≤ 0.1).

## Slide 6
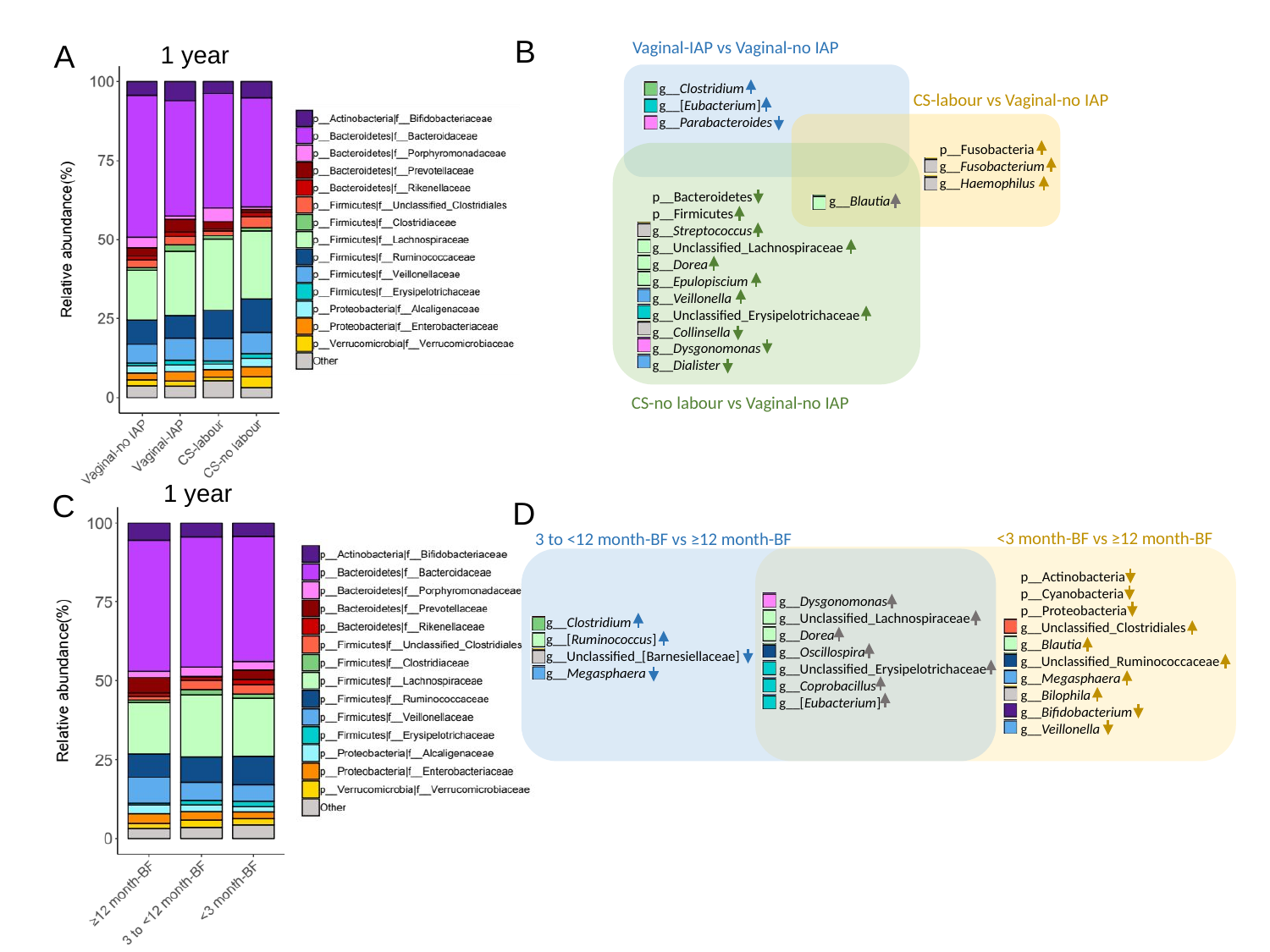

B
Vaginal-IAP vs Vaginal-no IAP
g__Clostridium
g__[Eubacterium]
g__Parabacteroides
CS-labour vs Vaginal-no IAP
p__Fusobacteria
g__Fusobacterium
g__Haemophilus
p__Bacteroidetes
p__Firmicutes
g__Streptococcus
g__Unclassified_Lachnospiraceae
g__Dorea
g__Epulopiscium
g__Veillonella
g__Unclassified_Erysipelotrichaceae
g__Collinsella
g__Dysgonomonas
g__Dialister
g__Blautia
CS-no labour vs Vaginal-no IAP
A
1 year
1 year
C
D
<3 month-BF vs ≥12 month-BF
3 to <12 month-BF vs ≥12 month-BF
p__Actinobacteria
p__Cyanobacteria
p__Proteobacteria
g__Unclassified_Clostridiales
g__Blautia
g__Unclassified_Ruminococcaceae
g__Megasphaera
g__Bilophila
g__Bifidobacterium
g__Veillonella
g__Dysgonomonas
g__Unclassified_Lachnospiraceae
g__Dorea
g__Oscillospira
g__Unclassified_Erysipelotrichaceae
g__Coprobacillus
g__[Eubacterium]
g__Clostridium
g__[Ruminococcus]
g__Unclassified_[Barnesiellaceae]
g__Megasphaera

## Slide 7
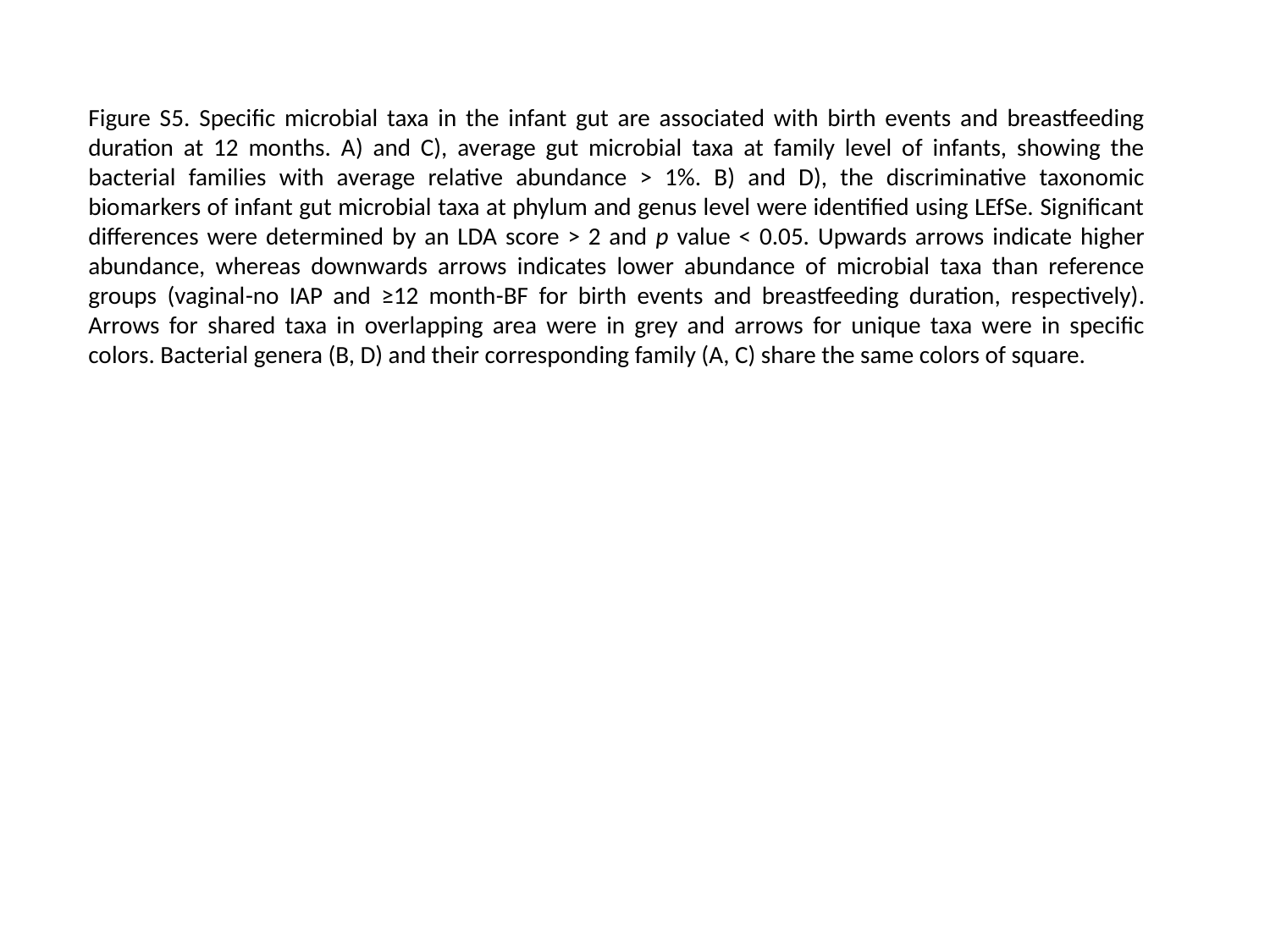

Figure S5. Specific microbial taxa in the infant gut are associated with birth events and breastfeeding duration at 12 months. A) and C), average gut microbial taxa at family level of infants, showing the bacterial families with average relative abundance > 1%. B) and D), the discriminative taxonomic biomarkers of infant gut microbial taxa at phylum and genus level were identified using LEfSe. Significant differences were determined by an LDA score > 2 and p value < 0.05. Upwards arrows indicate higher abundance, whereas downwards arrows indicates lower abundance of microbial taxa than reference groups (vaginal-no IAP and ≥12 month-BF for birth events and breastfeeding duration, respectively). Arrows for shared taxa in overlapping area were in grey and arrows for unique taxa were in specific colors. Bacterial genera (B, D) and their corresponding family (A, C) share the same colors of square.

## Slide 8
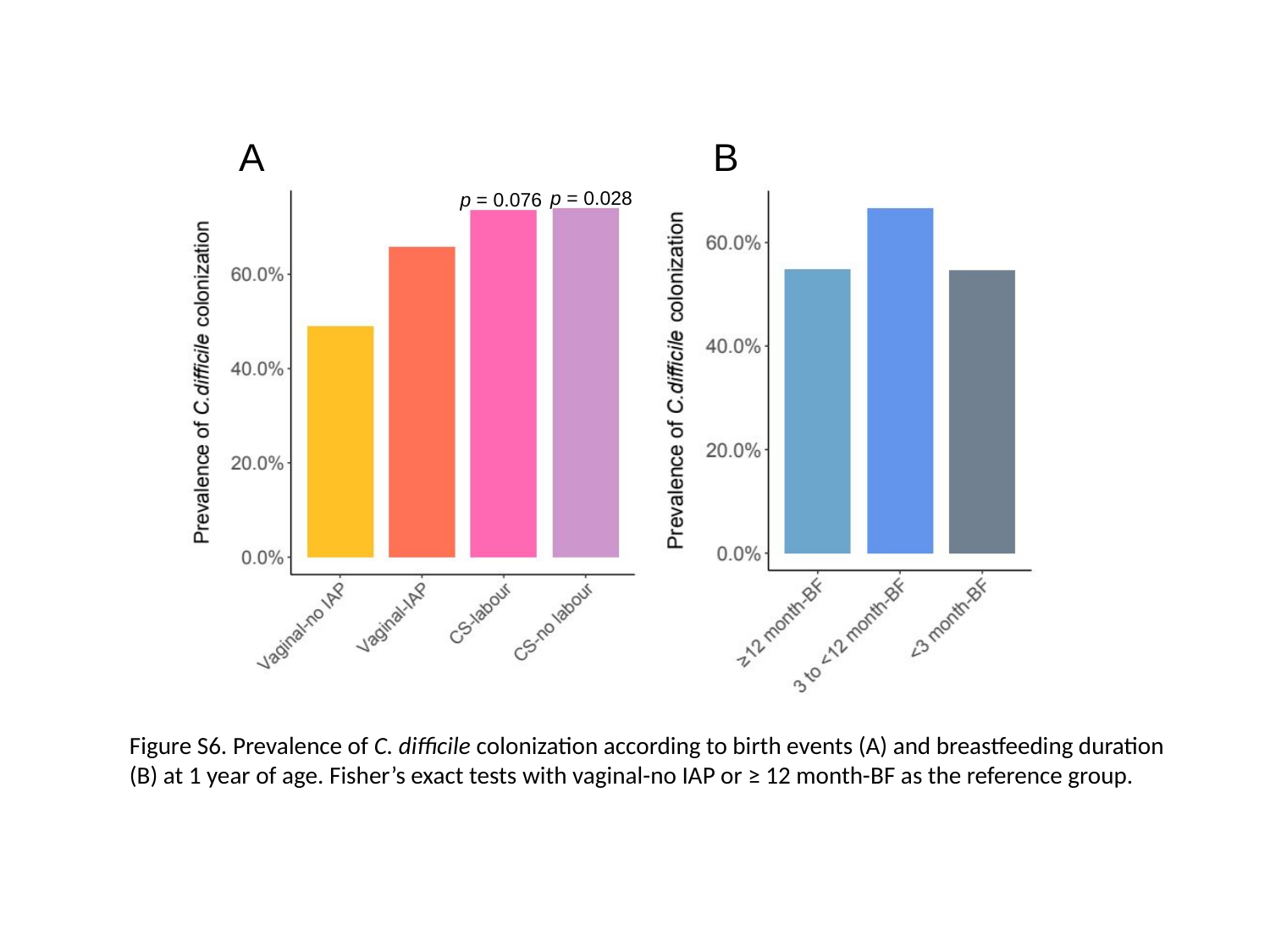

A
B
p = 0.028
p = 0.076
Figure S6. Prevalence of C. difficile colonization according to birth events (A) and breastfeeding duration (B) at 1 year of age. Fisher’s exact tests with vaginal-no IAP or ≥ 12 month-BF as the reference group.

## Slide 9
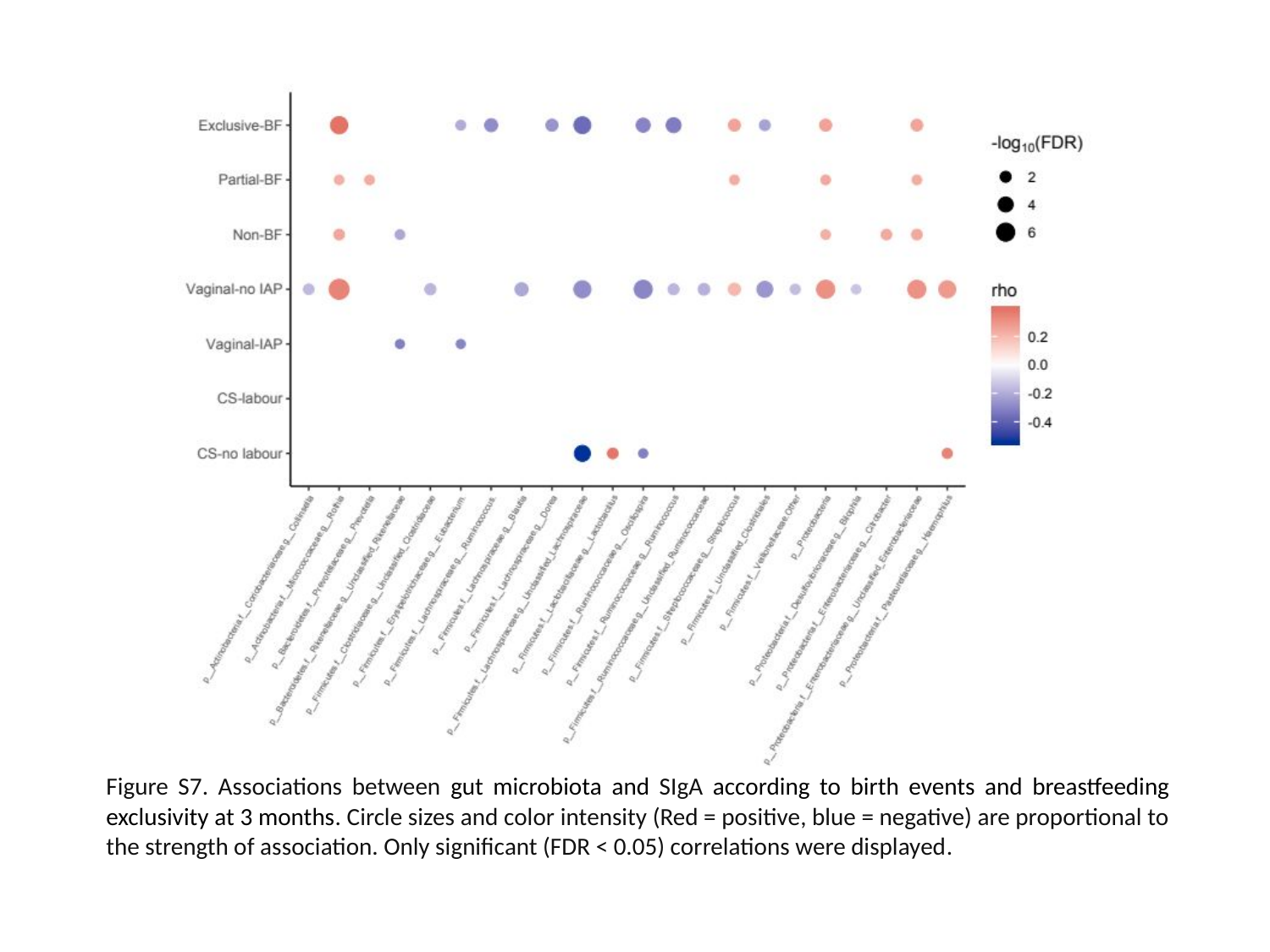

Figure S7. Associations between gut microbiota and SIgA according to birth events and breastfeeding exclusivity at 3 months. Circle sizes and color intensity (Red = positive, blue = negative) are proportional to the strength of association. Only significant (FDR < 0.05) correlations were displayed.

## Slide 10
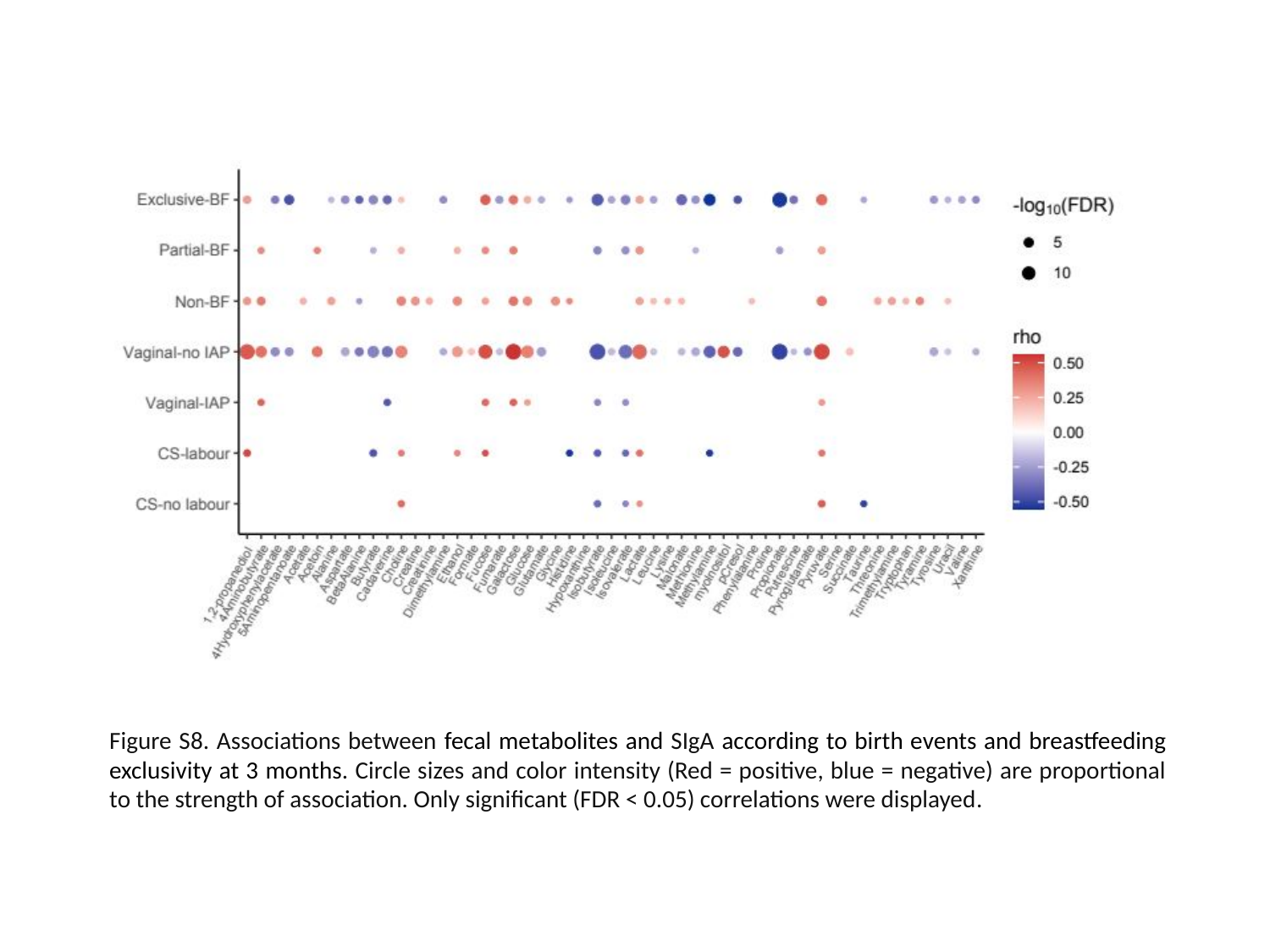

Figure S8. Associations between fecal metabolites and SIgA according to birth events and breastfeeding exclusivity at 3 months. Circle sizes and color intensity (Red = positive, blue = negative) are proportional to the strength of association. Only significant (FDR < 0.05) correlations were displayed.

## Slide 11
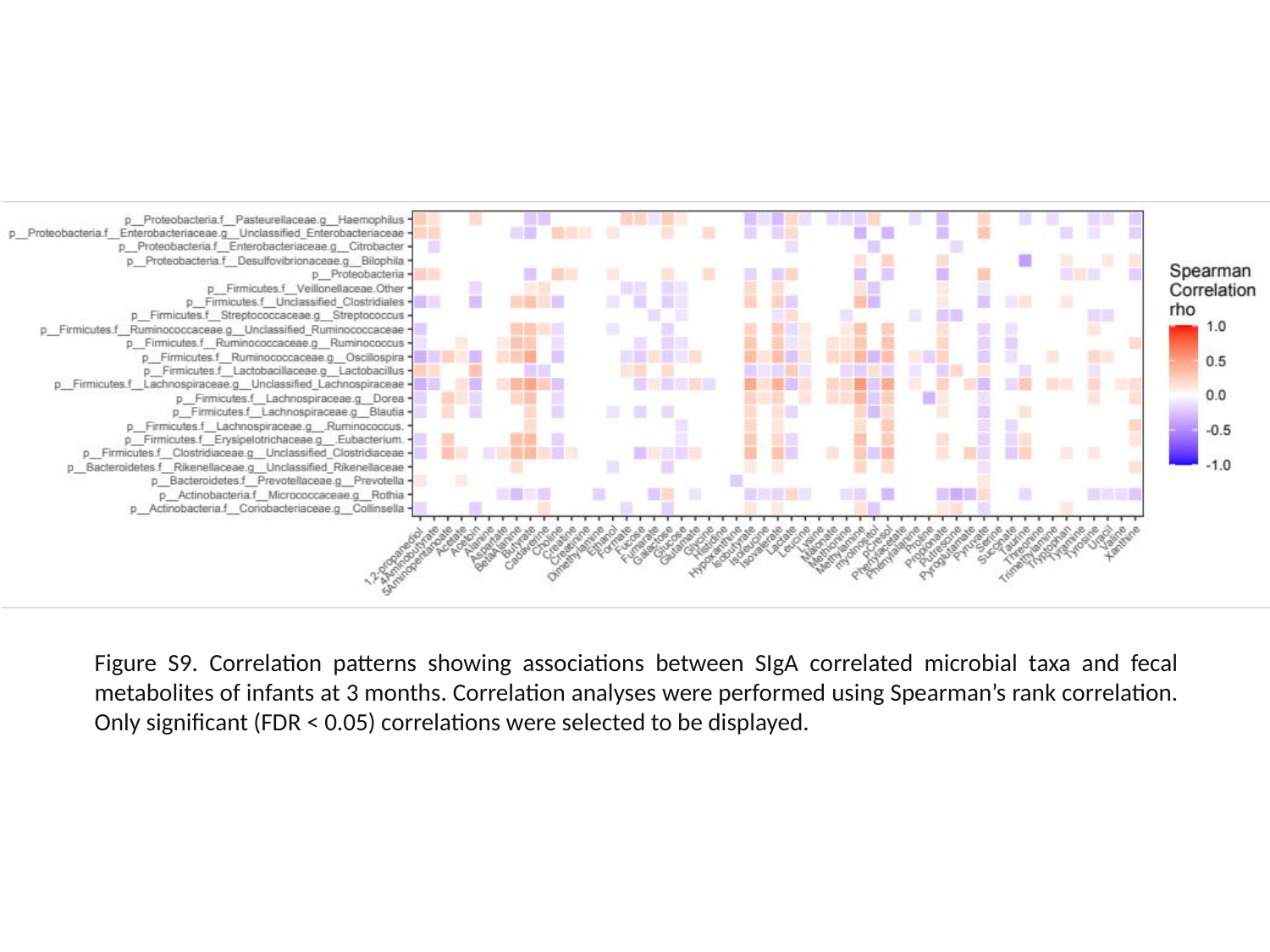

Figure S9. Correlation patterns showing associations between SIgA correlated microbial taxa and fecal metabolites of infants at 3 months. Correlation analyses were performed using Spearman’s rank correlation. Only significant (FDR < 0.05) correlations were selected to be displayed.

## Slide 12
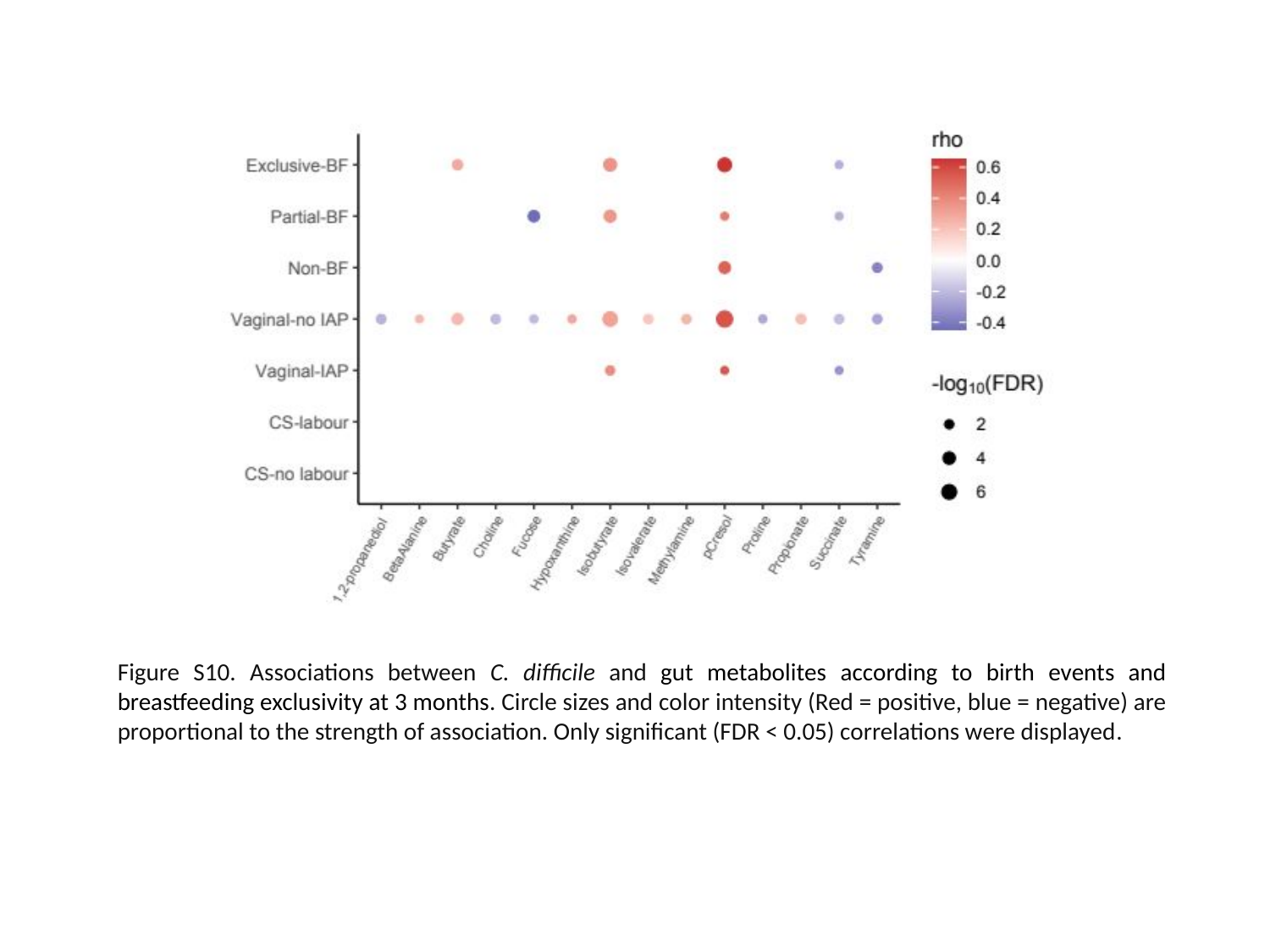

Figure S10. Associations between C. difficile and gut metabolites according to birth events and breastfeeding exclusivity at 3 months. Circle sizes and color intensity (Red = positive, blue = negative) are proportional to the strength of association. Only significant (FDR < 0.05) correlations were displayed.

## Slide 13
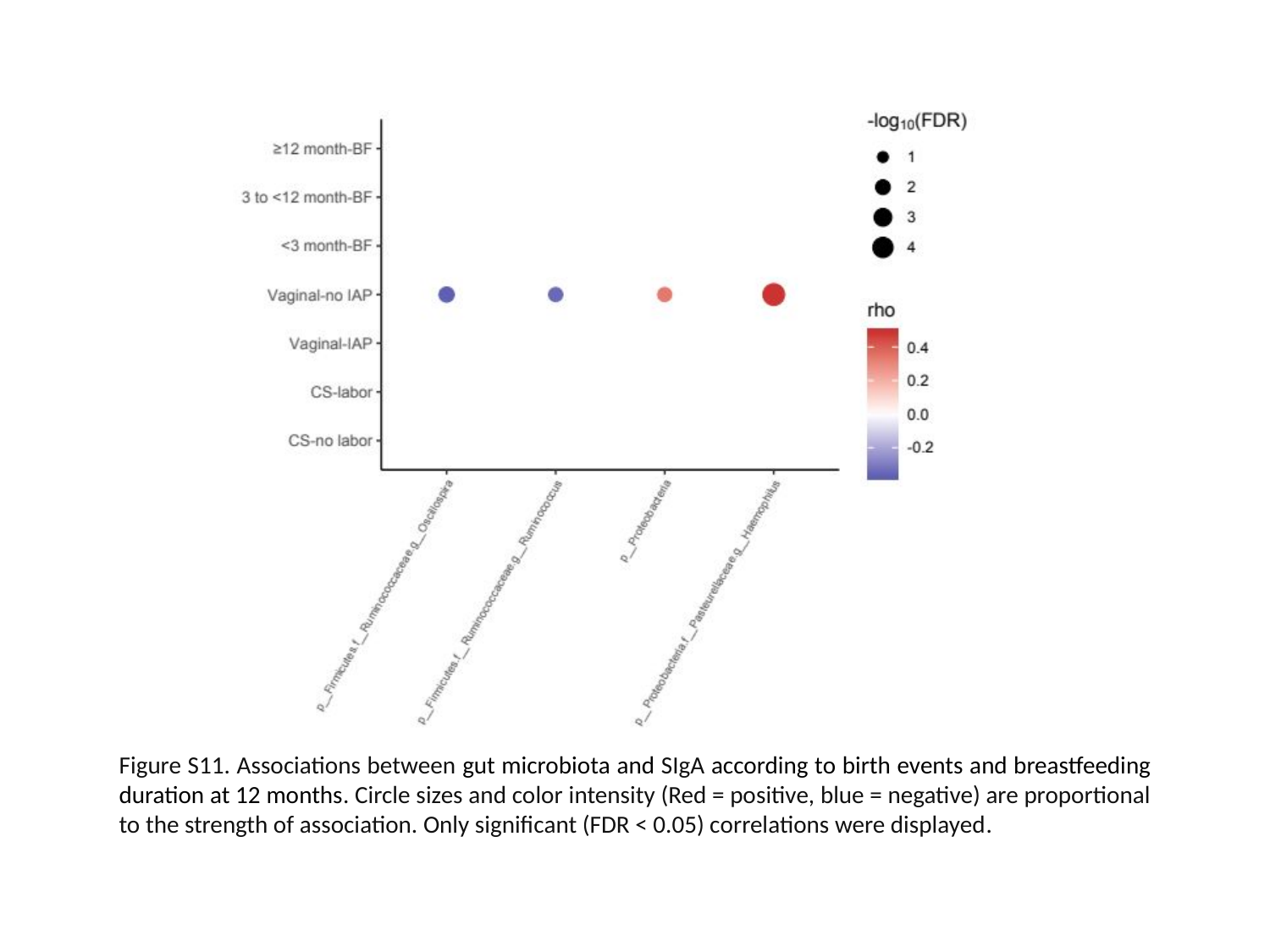

Figure S11. Associations between gut microbiota and SIgA according to birth events and breastfeeding duration at 12 months. Circle sizes and color intensity (Red = positive, blue = negative) are proportional to the strength of association. Only significant (FDR < 0.05) correlations were displayed.

## Slide 14
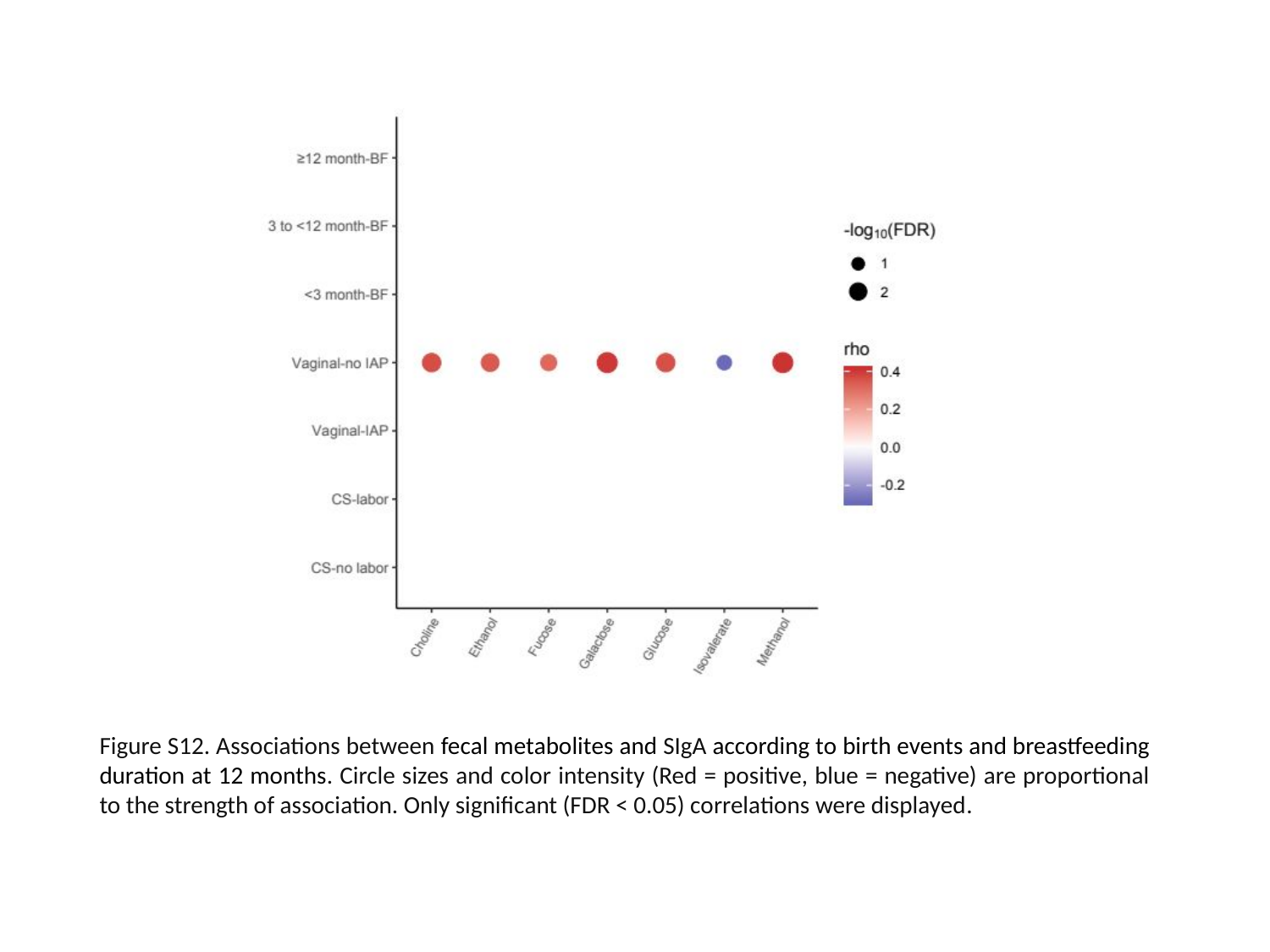

Figure S12. Associations between fecal metabolites and SIgA according to birth events and breastfeeding duration at 12 months. Circle sizes and color intensity (Red = positive, blue = negative) are proportional to the strength of association. Only significant (FDR < 0.05) correlations were displayed.

## Slide 15
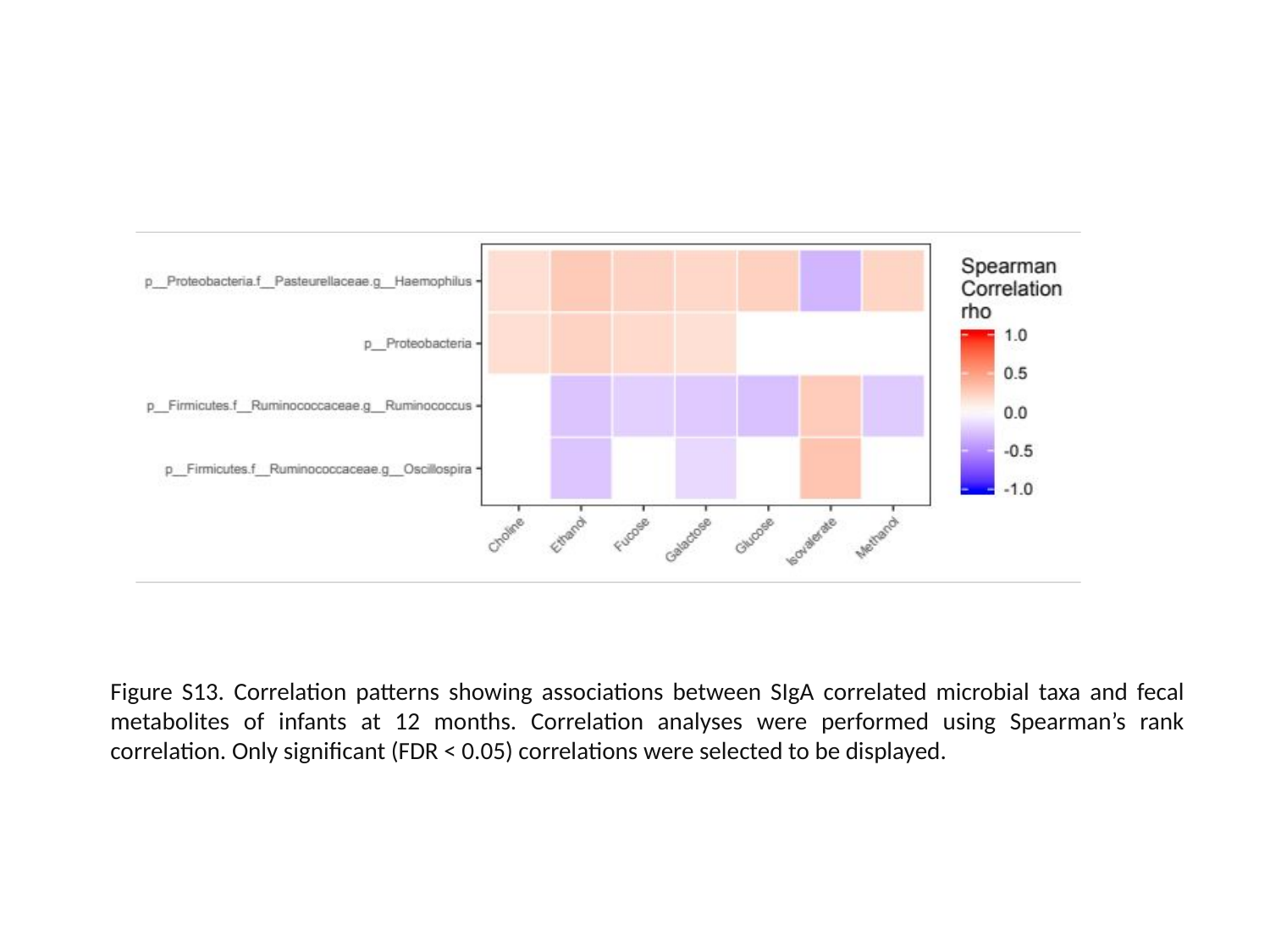

Figure S13. Correlation patterns showing associations between SIgA correlated microbial taxa and fecal metabolites of infants at 12 months. Correlation analyses were performed using Spearman’s rank correlation. Only significant (FDR < 0.05) correlations were selected to be displayed.
